# A rare overlap: an image of ANCA-associated glomerulonephritis superimposed on membranous nephropathy

**DOI:** 10.1590/2175-8239-JBN-2025-0308en

**Published:** 2026-03-16

**Authors:** Ana Catarina Oliveira, Maria Inês Vasconcelos Bessa, Roberto Silva

**Affiliations:** 1 Unidade Local de Saúde São João, Departamento de Nefrologia, Porto, Portugal.; 2 Unidade Local de Saúde São João, Departamento de Anatomia Patológica, Porto, Portugal.

**Figure 1:**
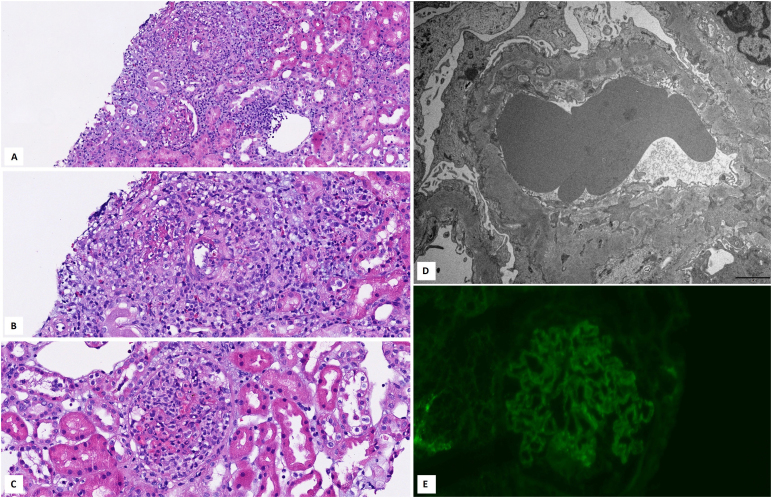
Kidney biopsy findings in a 77-year-old woman with MPO-ANCA-associated glomerulonephritis superimposed on membranous nephropathy. A: Glomerulus with a cellular crescent, and moderate interstitial infiltration by inflammatory cells (hematoxylin and eosin stain); B: Small artery with transmural fibrinoid necrosis and perivascular inflammation (hematoxylin and eosin stain); C: Glomerulus with a cellular crescent and fibrinoid necrosis (hematoxylin and eosin stain); D: Ultrastructural evaluation reveals subepithelial electron-dense deposits and foot process effacement; E: Immunofluorescence staining for IgG reveals granular global glomerular capillary wall positivity, typical of membranous nephropathy.

A 77-year-old woman with biopsy-proven membranous nephropathy (MN), diagnosed 14 years earlier with spontaneous remission, presented with fever, edema, and non-oliguric AKI (serum creatinine 1.1 mg/dL [baseline 0.6mg/dL], eGFR 52 mL/min/1.73 m^2^). Laboratory evaluation revealed leukocytosis, eosinophilia, proteinuria (1 g/24 h), RBC casts, positive anti-PLA2R (22 RU/mL), and elevated MPO-ANCA (149 U/mL). CT scan revealed chronic rhinosinusitis. Kidney biopsy showed crescents and fibrinoid necrosis in 4 of 47 glomeruli, 5 with global sclerosis, approximately 80% normal glomeruli, moderate eosinophilic interstitial infiltrate with perivascular inflammation, and 30% tubulointerstitial fibrosis. Immunofluorescence showed granular IgG and C3 deposition and electron microscopy confirmed subepithelial immune deposits consistent with MPO-ANCA-associated glomerulonephritis in the setting of EGPA, superimposed on MN (Figure 1), an exceptionally rare coexistence highlighting the need for long-term vasculitis surveillance in patients with MN^1^.
