# Supplementary material for: A rare overlap: an image of ANCA-associated glomerulonephritis superimposed on membranous nephropathy
Source: J Bras Nefrol. 2026 Mar 16;48(2):e20250308. doi: 10.1590/2175-8239-JBN-2025-0308en (PMC13007797; doi:10.1590/2175-8239-JBN-2025-0308en)
Supplement: Translation

# Translation: Uma rara sobreposição: uma imagem de glomerulonefrite ANCA associada à nefropatia membranosa

**DOI:** 10.1590/2175-8239-JBN-2025-0308pt

**Authors:** Ana Catarina Oliveira, Maria Inês Vasconcelos Bessa, Roberto Silva

**Affiliations:** 1 Unidade Local de Saúde São João, Departamento de Nefrologia, Porto, Portugal.; 2 Unidade Local de Saúde São João, Departamento de Anatomia Patológica, Porto, Portugal.

Uma mulher de 77 anos, diagnosticada com nefropatia membranosa (NM) por biópsia renal há 14 anos, com remissão espontânea, recorreu ao serviço de emergência por febre, edema e lesão renal aguda (LRA) não oligúrica (creatinina sérica 1,1 mg/dL [valor basal 0,6 mg/dL], TFGe 52 mL/min/1,73 m^2^). A avaliação laboratorial revelou leucocitose, eosinofilia, proteinúria (1 g/24 h), cilindros eritrocitários, anti-PLA2R positivo (22 RU/mL) e MPO-ANCA elevado (149 U/mL). A tomografia computadorizada revelou rinossinusite crônica. A biópsia renal revelou crescentes e necrose fibrinoide em 4 de 47 glomérulos, 5 com esclerose global, aproximadamente 80% de glomérulos normais, infiltrado intersticial eosinofílico moderado com inflamação perivascular e 30% de fibrose tubulointersticial. A imunofluorescência mostrou deposição granular de IgG e C3, e a microscopia eletrônica confirmou depósitos imunes subepiteliais, consistentes com glomerulonefrite associada à EGPA (MPO-ANCA positiva), sobreposta à NM (Figura 1), uma coexistência excepcionalmente rara que evidencia a necessidade de vigilância a longo prazo de vasculite em pacientes com NM^^1^^.

**Figura 1:** Biópsia renal de uma mulher de 77 anos com glomerulonefrite associada à MPO-ANCA sobreposta à nefropatia membranosa. A: Glomérulo com crescente celular e infiltração intersticial moderada por células inflamatórias (coloração por hematoxilina e eosina); B: Pequena artéria com necrose fibrinoide transmural e inflamação perivascular (coloração por hematoxilina e eosina); C: Glomérulo com crescente celular e necrose fibrinoide (coloração por hematoxilina e eosina); D: Microscopia eletrônica revela depósitos subepiteliais eletrondensos e apagamento dos pedicelos; E: A coloração por imunofluorescência para IgG revela positividade granular global na parede capilar glomerular, típica da nefropatia membranosa.
